# Inducible Prophage Mutant of *Escherichia coli* Can Lyse New Host and the Key Sites of Receptor Recognition Identification

**DOI:** 10.3389/fmicb.2017.00147

**Published:** 2017-02-01

**Authors:** Mianmian Chen, Lei Zhang, Sipei Xin, Huochun Yao, Chengping Lu, Wei Zhang

**Affiliations:** College of Veterinary Medicine, Nanjing Agricultural UniversityNanjing, China

**Keywords:** prophage, *Escherichia coli*, tail fiber protein, receptor recognition, host range expansion

## Abstract

The use of bacteriophages as therapeutic agents is hindered by their narrow and specific host range, and by a lack of the knowledge concerning the molecular mechanism of receptor recognition. Two P2-like coliphages, named P88 and pro147, were induced from *Escherichia coli* strains K88 and DE147, respectively. A comparison of the genomes of these two and other P2-like coliphages obtained from GenBank showed that the tail fiber protein genes, which are the key genes for receptor recognition in other myoviridae phages, showed more diversity than the conserved lysin, replicase, and terminase genes. Firstly, replacing hypervariable region 2 (HR2: amino acids 716–746) of the tail fiber protein of P88 with that of pro147 changed the host range of P88. Then, replacing six amino acids in HR2 with the corresponding residues from pro147 altered the host range only in these mutants with changes at position 730 (leucine) and 744 (glutamic acid). Thus, we predicted that these amino acids are vital to establish the host range of P88. This study provided a vector of lysogenic bacteria that could be used to change or expand the phage host range of P88. These results illustrated that, in P2-like phage P88, the tail fiber protein determined the receptor recognition. Amino acids 716–746 and the amino acids at positions 730 and 744 were important for receptor recognition.

## Introduction

*Escherichia coli* is an important pathogen in intestinal and extraintestinal infections (Johnson and Russo, 2002; Kaper et al., 2004; Kim, 2012; Croxen et al., 2013). Antibiotics are commonly used to treat colibacillosis (infection with a bacteria called *E. coli*); however, antibiotic resistance among bacteria, especially that of multi-drug-resistant strains (Kim et al., 2007; Hasan et al., 2011; Liu et al., 2016) have led to the reconsideration of bacteriophages as alternative therapeutic agents (Brussow, 2012; Keen, 2012; Young and Gill, 2015; Gorski et al., 2016). Compared with traditional antibiotic therapy, phages can target specific bacteria and reduce damage to the normal flora of the host (Clark and March, 2006). Unfortunately, the main drawback of phages as therapeutic agents for bacterial infection is their narrow host range (Haq et al., 2012).

In general, phages can undergo either a lytic or lysogenic lifecycle, and the genome of a lysogenic phage is integrated into the host chromosome and replicated passively as the host genome is replicated. Lysogenic phages can exist as phage particles and as part of the host genome. When in the host chromosome, the phage genome can be modified by manipulating the bacterial genome using molecular biological techniques (Posfai et al., 1999; Datsenko and Wanner, 2000; Yu et al., 2008; Blank et al., 2011).

In the *Myoviridae* adsorption process, such as that of phage T4, binding to cell membrane surfaces during the infection processes has two steps: first, specific host recognition occurs through a reversible interaction of the tail fibers with lipopolysaccharides or with the outer membrane porin protein C (Yu and Mizushima, 1982; Bartual et al., 2010); second, the tail spikes (also called short tail fibers) extend and irreversibly bind to the lipopolysaccharides (Riede, 1987; Thomassen et al., 2003). The host selectivity of a phage is determined by its tail fiber specificity, meanwhile tail spike specificity is as important for the infection process and host recognition as tail fiber specificity (Kageyama et al., 2009). Tail fiber proteins of many phages have been identified as important regions for receptor recognition determination experimentally (Riede et al., 1985; Montag et al., 1990; Yoichi et al., 2005; Mahichi et al., 2009; Bartual et al., 2010). In P2 like phages, only the tail spike has been proven to be the important region for the receptor recognition (Kageyama et al., 2009), while the involvement of the tail fiber protein has not been proven experimentally. Thus, in the present study, we isolate P2-like phages and determined whether the tail fiber protein was the receptor recognition region. The different parts of the tail fiber of one phage were replaced with the corresponding parts of another phage, and then the host ranges of the phage mutants were analyzed. We aimed to identify the specific binding sequences that determine the phage host range and to alter or expand the phage host range in the vector of lysogenic bacteria.

## Materials and Methods

### Bacterial Strains, Plasmids, and Growth Conditions

Strains used for prophage induction and phage isolation were listed in **Supplementary Tables S1** and **S2**, respectively. Strain K88 came from a swine source. In addition, the 101 avian pathogenic *E. coli* strains used in these experiments were isolated from the brains of ducks with clinical signs of septicemia and neurological symptoms at different times and in different areas in the east of China, as previously described (Ma et al., 2013) (**Supplementary Table S2**). All *E. coli* strains were grown in Luria-Bertani (LB) medium at 37°C. When necessary, the LB medium was supplemented with appropriate antibiotic: ampicillin (100 μg/mL), kanamycin (50 μg/mL), chloramphenicol (30 μg/mL), or tetracycline hydrochloride (5 μg/mL). The plasmids used in this study were listed in **Supplementary Table S3**. The Red- and I-SceI-expressing plasmid pWRG99 was a derivative of pKD46 (Datsenko and Wanner, 2000; Blank et al., 2011).

### Prophage Induction from the Chromosome of 54 *E. coli* Strains

Bacteriophages were obtained following mitomycin C (Sigma, St. Louis, MO, USA) induction of 54 lysogenic strains (**Supplementary Table S1**). Each strain was cultured for 5 h in LB medium and then diluted 1:100 in 5 mL of fresh medium. When the optical density at 600 nm (OD600) of the cultures reached 0.2, mitomycin C was added to a final concentration of 500 ng/mL. The cultures were then incubated at 37°C for 10 h. The lysates were centrifuged for 10 min at 3,500 × *g* at 4°C, and the supernatants were collected for follow-up experiments. The double-layer agar plate method (Adams, 1959) was used to find the host of the induced bacteriophage from the 108 *E. coli* strains (**Supplementary Table S2**).

### Optimal Multiplicity of Infection (MOI) and One-Step Growth Curve of P88

#### Optimal MOI

Early log phase cells were infected with P88 at five different ratios (0.1, 0.01, 0.001, 0.0001, and 0.00001). After incubation for 3.5 h at 37°C, the phage lysates was centrifuged at 3,500 × *g* for 5 min. The supernatant was diluted to determine the phage titer (Lu et al., 2003).

#### One-Step Growth Curve

The phage and bacteria were mixed. Samples only containing absorbed phage were taken at 5 min intervals and then diluted to determine the plaque forming units (pfu) using the double-layer plate method (Chow et al., 1988; Ma and Lu, 2008; Hejnowicz et al., 2009).

### Electron Microscopy

The phage filtrate was applied to a copper grid before negative staining with phosphotungstic acid (PTA, 2% w/v). Electron micrographs were observed using an H_7650 transmission electron microscope (TEM; Hitachi, Japan).

### Phage DNA Extraction

Phage DNA extraction was performed as previously described (Ma and Lu, 2008) with some modifications. The phage pellet was digested with 2 μg/mL DNase and 10 μg/mL RNase for 30 min at 37°C. Phages were purified with NaCl-polyethylene glycol (PEG) 8000, and the DNA was isolated using SDS-Proteinase K. To analyze the quality of the DNA fragments, the molecules were separated by 0.8 % (w/v) agarose gel electrophoresis in TAE buffer (40 mM Tris-HCl, 500 mM sodium acetate, 50 mM EDTA, pH 7.2). The DNA was then suspended in ultrapure water.

### Genome Sequencing

The lysogenic bacteria K88, host bacteria DE048, and phage P88 were analyzed by high-throughput sequencing to confirm if the phage P88 genome is located in the K88 genome. The phage P88 and pro147 genomes were sequenced by Roche 454 Sequencing, and reads were assembled into contigs using the Newbler (version 2.8). Strain K88 and the DE048 genomes were sequenced by Illumina MiSeq Sequencing. Reads were assembled into contigs using the Newbler.

### Functional Analysis of Phage P88 and pro147

RAST (Aziz et al., 2008; Overbeek et al., 2014; Brettin et al., 2015) was used to predict the open reading frames (ORFs) of sequences and to analyze their corresponding functions. BLASTp was used to analyze the putative ORFs against the NCBI non-redundant proteins (NR) database. The phylogenetic trees of several selected genes were constructed with MEGA5.2 (Tamura et al., 2011) using the Neighbor-Joining algorithm. The structural model was generated using the Phyre^1^ server^1^ (Kelley et al., 2015).

### Comparative Genome Analysis

Nucleic acid sequences of 10 P2-like phages [P88, pro147, P2 (NC_001895.1), Wphi, PsP3, 186, L-413C, Fels-2, fiAA91-ss, and P2 (KC618326.1)] were downloaded from GenBank. Comparisons of nucleic acid sequences were carried out with BLASTn. Comparisons of nucleic acid sequences of P88 (or pro147) with other P2-like phages were performed. Comparisons of nucleic acid sequences of P88 with pro147 were also performed. Phylogenetic trees based on the amino acid sequences (the lysins, the replicase, the tail fiber protein and the ATPase subunit of the terminase) were constructed with MEGA to analyze their evolutionary relationships.

### Comparison of Tail Fiber Amino Acid Sequences

Amino acid sequences of the tail fiber proteins of 10 P2-like phages [P88, pro147, P2 (NC_001895.1), Wphi, PsP3, 186, L-413C, Fels-2, fiAA91-ss, and P2 (KC618326.1)] were downloaded from the NCBI database and comparisons of amino acid sequences were carried out with ClustalX (version 2.1). The phylogenetic tree of the tail fiber proteins showed that P88 had a close evolutionary relationship with pro147, P2 (NC_001895.1), and Wphi. The amino acid sequences of the tail fiber proteins of P88, pro147, P2 (NC_001895.1), and Wphi were then compared to analyze which segments of encoding DNA could be selected for gene manipulation. The amino acid sequences of the tail fiber proteins of P88 and pro147 were compared to select the replacement sections.

### Construction of Gene Mutants to Analyze the Key Regions and Sites

The recombineering method of scarless mutagenesis was carried out according to the protocol described previously (Blank et al., 2011) with some modifications. The recombineering method of scarless mutagenesis was divided into two steps. In the first step, the target gene was replaced with a chloramphenicol resistance cassette (cat) gene and the I-SceI recognition site, which was PCR-amplified from plasmid pKD3 (**Supplementary Table S3**). The primers (**Supplementary Table S3**) were homologous to the flanking region of the corresponding target gene. The PCR products were then transformed by electroporation into K88 containing the lambda Red recombinase expression plasmid pKD46 (Datsenko and Wanner, 2000; Wang et al., 2011). After electroporation, samples were incubated at 30°C for 90 min in SOC broth (Datsenko and Wanner, 2000) and plated on LB agar with chloramphenicol to select for target gene mutants. Mutants were confirmed by PCR and sequencing using primers k1 and k2 (c1 and c2) (**Supplementary Table S3**). In the second step, the PCR products containing regions homologous to the flanking regions of the resistance gene for homologous recombination were transformed by electroporation into K88 containing the lambda Red recombinase expression and I-SceI endonuclease expression plasmid pWRG99 (**Supplementary Table S3**). After electroporation, samples were incubated at 30°C for 90 min in SOC broth and plated on LB agar with tetracycline hydrochloride. Selection of successful recombinants was mediated by the sequential expression of I-SceI endonuclease after Red-mediated recombination. If recombination occurred, the 200–500 bp dsDNA fragment harboring the same homologous region would replace the corresponding sequence of K88. If no recombination occurred, the unique I-SceI restriction site within the genome would still be present and the I-SceI endonuclease expressed from plasmid pWRG99 would kill unsuccessful recombinants by induction of double-strand breaks. Finally, pWRG99 was cured by streaking colonies and incubated at 37°C. Mutants were confirmed by PCR and sequencing using primers check1 and check2 (**Supplementary Table S3**).

### Induction, Lytic Capacity Analysis, and Host Range Test of P88 Mutants

Each K88 mutant strain was cultured for 5 h in LB medium and then diluted 1:100 in 5 mL fresh medium. When the culture reached an OD600 of 0.2, mitomycin C was added and then was incubated at 30°C for 10 h. The lysates were centrifuged and used to test their lytic capacity by the double-layer agar plate method with DE048, the host of P88.

Phage P88 could not infect the bacteria if the phage suspension was dropped onto the LB agar plate spread with bacteria, so the host range analysis of each P88 mutant strain constructed by the scarless mutagenesis method was carried out by the double-layer agar plate method with DE048, MC1061, DH5a and BL21.

## Results

### Two Prophages of P88 and pro147 Were Induced from 54 *E. coli* Strains

**FIGURE 1:**
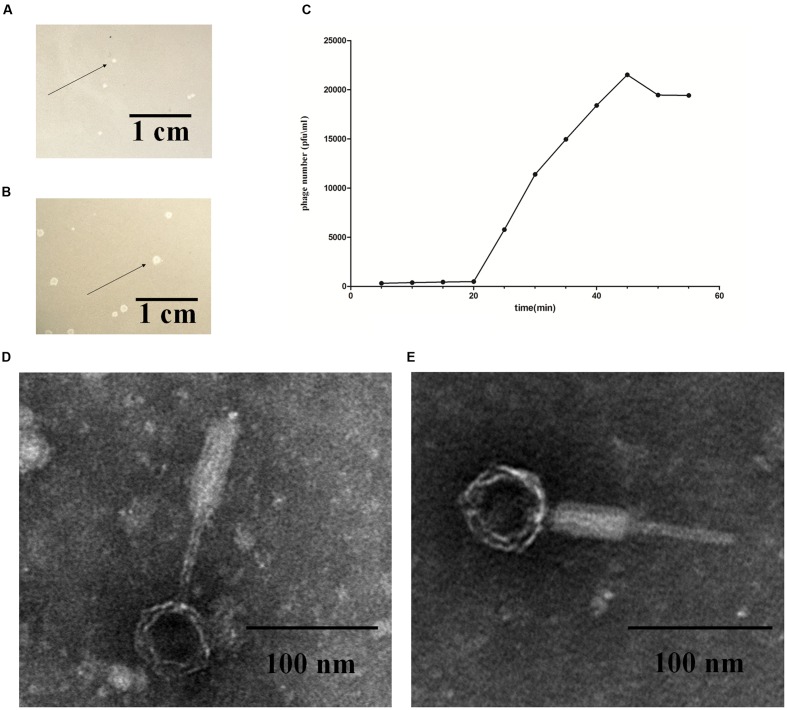
**Fundamental characteristics of P2-like phages.** **(A)** Plaques formed by phage P88. **(B)** Plaques formed by phage pro147. Growth of the phages (P88 and pro147) on *Escherichia coli* DE048 in double agar LB plates for 6 h. P88 formed clear and round plaques of approximately 0.06 cm in diameter. Pro147 formed clear and round plaques of approximately 0.13 cm in diameter. **(C)** One-step growth curve. Phage and bacteria were mixed, and samples containing only absorbed phage were taken at 5 min intervals and diluted. The double-layer plate method was used to determine the plaque forming units (pfu). One-step growth curve for P88 showed a latent period of about 20 min, a rise period of 25 min, and an average burst size of about 65. **(D)** Electron microscopy image of P88. **(E)** Electron microscopy image of pro147. Electron microscopy showed that the heads of P88 and pro147 were icosahedrons, and the tails was long and could shrink. For P88, the diameter of its capsid head was estimated at 50 nm and the tail length was estimated at 140 nm. For pro147, the diameter of its capsid head was estimated at 50 nm and the tail length was estimated at 130 nm.

Phage induction of 54 *E. coli* strains was investigated by the addition of mitomycin C. After induction, cell lysates were prepared and tested for their ability to lyse various *E. coli* hosts in plaque assays using the 108 strains listed in **Supplementary Table S2**. Out of the 54 *E. coli* strains used, the lysates of *E. coli* strain K88 and DE147 produced phages that could lyse the clinical isolates of avian pathogenic *E. coli* (APEC) strain DE048. These two phages were designated as P88 and pro147. After growth on DE048 in double agar LB plates at 37°C for 12 h, P88 formed clear and round plaques of approximately 0.06 cm in diameter (**Figure 1A**); pro147 formed clear and round plaques of approximately 0.13 cm in diameter (**Figure 1B**). Among the 108 *E. coli* strains tested, P88 specifically lysed DE048, while pro147 specifically lysed DE048, DH5a, BL21, and MC1061.

### Optimal MOI and One-Step Growth Curve of P88

After incubation for 3.5 h, P88 had the highest concentration (1.1 × 10^10^) at the ratio of 0.01 (pfu/cfu); therefore, the ratio of 0.01 (pfu/cfu) was considered as the optimal MOI. The one-step growth curve obtained for P88 showed a latent period of about 20 min, a rise period of 25 min, and an average burst size of about 65 (**Figure 1C**).

### Electron Microscopy of P88 and pro147

Electron microscopy showed that the heads of P88 and pro147 were icosahedrons, and the tail was long and could shrink. In P88, the diameter of the capsid head was estimated at 55 nm and the tail length was estimated at 137 nm (**Figure 1D**). For pro147, the diameter of the capsid head was estimated at 54 nm and the tail length was estimated at 131 nm (**Figure 1E**). Phage P88 and pro147 belong to *Myoviridae* phages.

### Genome Sequencing and Analysis

#### Genome Submission

P88 and pro147 genomes were submitted to GenBank with the accession numbers NC_026014.1 and NC_028896.1, respectively. The Whole Genome Shotgun project of DE048 and K88 were deposited at DDBJ/EMBL/GenBank under the accession numbers LBBM00000000 and LBBN00000000, respectively.

#### Overview of the P88 and pro147 Genomes

**FIGURE 2:**
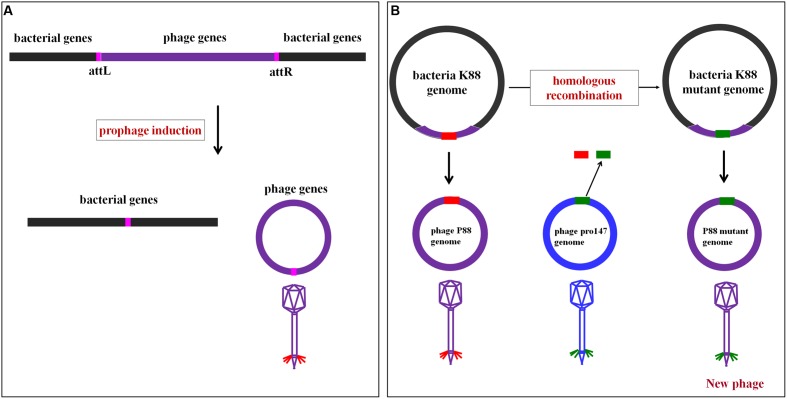
**Mechanism of P88 prophage genome excision from the *E. coli* K88 genome and the rationale of homologous recombination of P88.** **(A)** Mechanism of P88 prophage genome excision from the *E. coli* K88 genome. Schematic representation of part of the lysogenic bacteria K88 genome and the location of the attL and attR regions. During excision, the P88 genome is circularized between the flanking host chromosomes. **(B)** The rationale of homologous recombination of P88. The phage genome is modified in the genome of lysogenic bacteria and then prophage production is induced. First, the target gene of P88 was selected for to manipulation, and then the segments of target gene were replaced with the corresponding segments of pro147 which were transformed by electroporation into K88. Finally, K88 mutant prophages were induced.

Comparative genome analysis showed that the P88 genome came from the *E. coli* K88 genome, and the mechanism of P88 prophage genome excision from *E. coli* K88 genome was displayed in **Figure 2A**. The P88 genome consisted of a double-stranded 35,814-bp DNA with 53 putative ORFs. The attP site of the P88 genome was determined as a 14-bp fragment (GCCACCCGAAGGTG). The pro147 genome consisted of a double-stranded 32,675-bp DNA with 44 putative ORFs. BLAST analysis of the P88 sequence at the NCBI database revealed that it had high similarity to prophages in *E. coli, Shigella boydii, and Salmonella enterica*, with more than 76 strains having coverage higher than 68% (ident 96%). However, P88 had low homology with reported P2-like phages and shared low similarity with P2, covering 5% (ident 90%).

BLAST analysis of the pro147 sequence at the NCBI database revealed that it had high similarity to the prophages in *E. coli, S. sonnei, Klebsiella pneumonia*, and *S. enterica*, with more than 68 strains having coverage higher than 68% (ident 96%). Phage pro147 had high similarity to the reported P2-like phages [P2: coverage 75% (ident 97%); Wphi: coverage 75% (ident 98%); L-413C: coverage 73% (ident 96%); fiAA91-ss: coverage 73% (ident 98%)]. Although both P88 and pro147 could infect *E. coli* strain DE048, the P88 genome exhibited low similarity with pro147, covering 6% (ident 92%).

#### Functional Module Analysis

**FIGURE 3:**
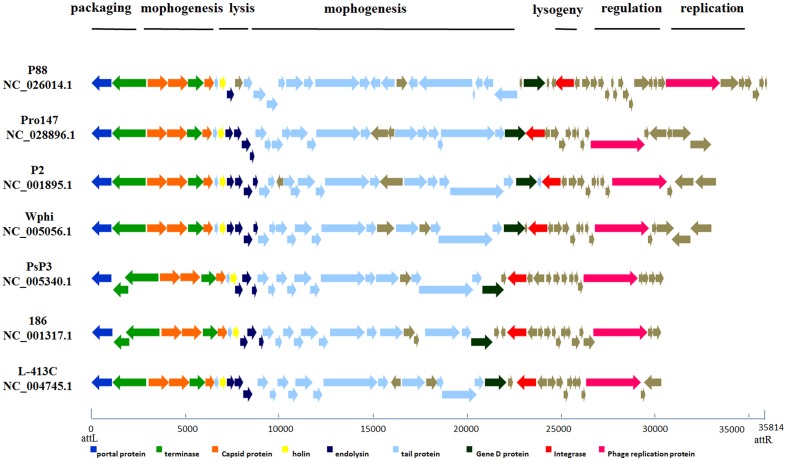
**Comparative genome alignments of the seven P2-like phages with similar modules.** Modular organization of seven P2-like phage genomes [P88, pro147, P2 (NC_001895.1), Wphi, PsP3, 186 and L-413C]. Genes are grouped into six functional modules associated with the phage life cycle: lysogeny, replication, regulation, packaging, morphogenesis, and lysis. Sets of genes with similar function are grouped using the same color. The bottom line provides a base pair scale of the genomes.

According to the putative functions of the ORFs predicted by RAST and BLASTp analyses, the P88 genome could be organized into six major functional modules involved in morphogenesis, replication, regulation, packaging, lysogeny, and lysis. Functional module and comparative genome analysis demonstrated that P88 and six other P2-like phages [P2 (NC_001895.1), Wphi, PsP3, 186, L-413C, and pro147] had similar functional modules (**Figure 3**).

#### Evolutionary Relationship Analysis

Phylogenetic trees based on the amino acid sequences (the lysins, the replicase, the tail fiber protein, and the ATPase subunit of the terminase) of the 10 P2 like phages [P88, pro147, P2 (NC_001895.1), Wphi, PsP3, 186, L-413C, Fels-2, fiAA91-ss, and P2 (KC618326.1)] were constructed. These 10 prophage genomes, which were P2-like phages whose hosts belong to the *Enterobacteriaceae*, were obtained from the NCBI database (**Table 1**).

**Table 1:** Properties of P2-like phages whose hosts belong to the *Enterobacteriaceae.*

Phage	host	Host from	Accession no.	Genome size (bp)	%GC	Coding Sequences
Enterobacteria phage P2	*Escherichia coli*	Sweden	NC_001895.1	33593	50.2	43
Enterobacteria phage P2	*Escherichia coli* O157:H43 str. T22	Hungarian	KC618326.1	31200	52.6	46
Bacteriophage 186	*Escherichia coli*	Australia	NC_001317.1	30624	53.1	46
Bacteriophage Wphi	*Escherichia coli*	USA	NC_005056.1	32684	51.7	44
Enterobacteria phage fiAA91-ss	*Escherichia coli* O157:H7	Spain	NC_022750.1	33628	51.9	40
Enterobacteria phage Fels-2	*Salmonella* typhimurium LT2	USA	NC_010463.1	33693	52.5	47
Enterobacteria phage PsP3	*Salmonella*	USA	NC_005340.1	30636	52.8	42
Yersinia phage L-413C	*Yersinia pestis*	USA	NC_004745.1	30728	52.1	40
Enterobacteria phage P88	*Escherichia coli* K88	China	NC_026014.1	35814	52.9	53
Enterobacteria phage pro147	*Escherichia coli* DE147	China	NC_028896.1	32675	50.7	44


**FIGURE 4:**
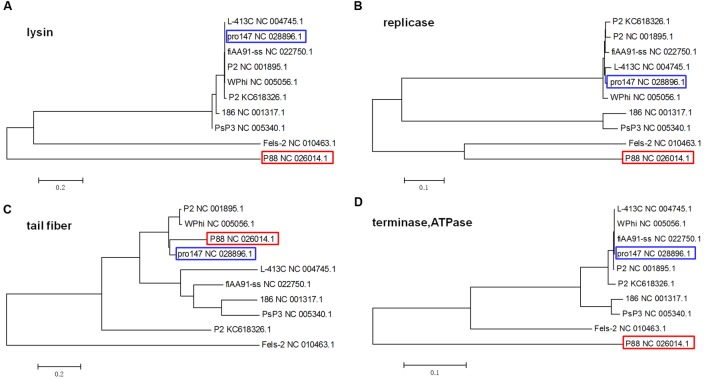
**Phylogenetic trees (the lysins, the replicase, the tail fiber protein, and the ATPase of the terminase) of the 10 P2-like phages whose hosts belong to the *Enterobacteriaceae*.** Neighbor-joining tree analysis and bootstrap analysis (500 bootstrap Replications) based on the alignment of the amino acid sequence of the lysins **(A)**, the replicase **(B)**, the tail fiber protein **(C)**, and the ATPase subunits **(D)** of the 10 P2-like phages [P88, pro147, P2 (NC_001895.1), Wphi, PsP3, 186, L-413C, Fels-2, fiAA91-ss, and P2 (KC618326.1)]. The numbers at the nodes indicate the bootstrap probabilities of that particular branch.

The phylogenetic trees based on the amino acid sequences of lysin, replicase and terminase (ATPase subunit) showed that phage P88 appeared to be phylogenetically distinct from the other P2-like phages, while, pro147 had close evolutionary relationships with P2 (NC_001895.1), Wphi, L-413C, and fiAA91-ss (**Figures 4A,B,D**). In the phylogenetic tree of the tail fiber protein, P88 had a close evolutionary relationship with pro147, P2 (NC_001895.1) and Wphi (**Figure 4C**). These results showed that the genes of tail fiber proteins were more diverse than the conserved genes of lysin, replicase and terminase.

### Comparison of Tail Fiber Amino Acid Sequences

**FIGURE 5:**
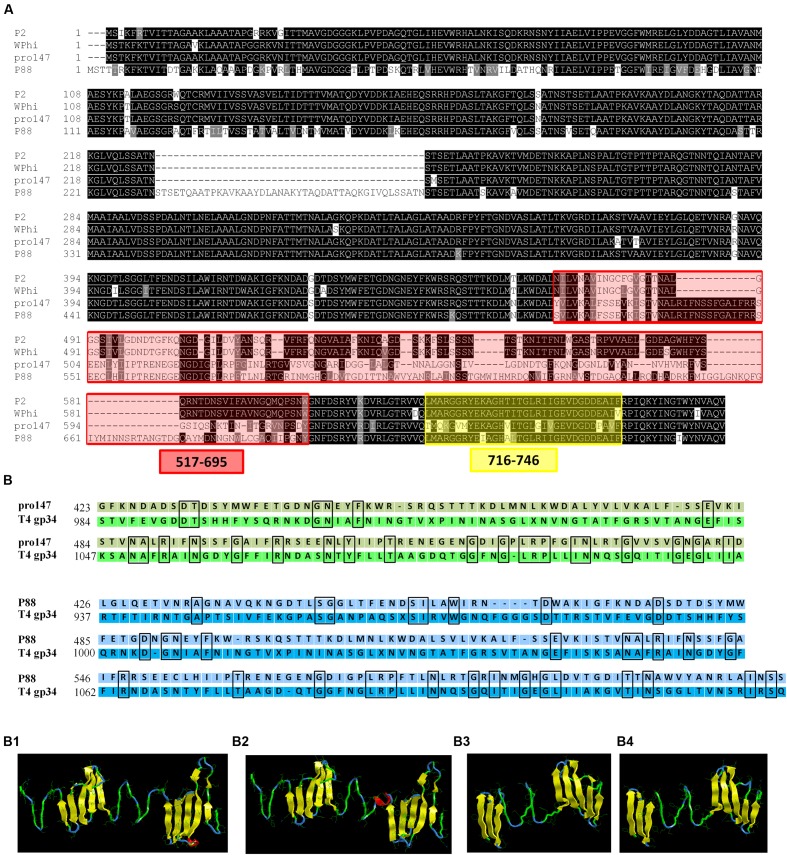
**Alignment of the tail fiber structural proteins and structure prediction and modeling of the tail fiber proteins of P88 and pro147.** **(A)** Alignment of the P88 tail fiber protein with similar regions (tail fiber proteins) from phages pro147, P2 (NC_001895.1) and Wphi. The tail fiber protein sequence of P88 shared high similarity with that of pro147, covering 100% (ident 70%); P2 (NC_001895.1), covering 100% (ident 65%), and Wphi, covering 100% (ident 62%). There were two hypervariable regions of 178aa (517–695 in P88) and 31 aa (716–746 in P88), respectively, in the C-terminal region. **(B)** Structure prediction and modeling of the tail fiber proteins of P88 and pro147. Sequence alignment of phage P88 tail fiber (or pro147) with its homologs from *E. coli* phage T4 gp34 were processed. **(B1)** The model crystal structure of amino acids 937–1127 of T4 gp34 (PDB code: c4uxeB.1). **(B2)** Homology modeling of P88 tail fiber based on the crystal structure of amino acids 937–1127 of T4 gp34 (PDB code: c4uxeB.1). The model was generated using the Phyre2 server (http://www.sbg.bio.ic.ac.uk/phyre2/html/). **(B3)** The model crystal structure of amino acids 984–1108 of T4 gp34 (PDB code: c4uxeB.1). **(B4)** Homology modeling of pro147 tail fiber based on the crystal structure of amino acids 984–1108 of T4 gp34 (PDB code: c4uxeB.1). The model was generated using the Phyre2 server (http://www.sbg.bio.ic.ac.uk/phyre2/html/).

The P88 genome exhibited low similarity with pro147, covering 6% (ident 92%); however, the tail fiber protein sequence of P88 shared high similarity with that of pro147, covering 100% (ident 70%). In addition, the P88 tail fiber protein shared high similarity with that of P2 (NC_001895.1), covering 100% (ident 65%) and Wphi, covering 100% (ident 62%). Alignment of the P88 tail fiber protein with those of pro147, P2 and Wphi showed that there were two hypervariable regions of 178aa (517–695 in P88) and 31 aa (716–746 in P88) in the C-terminal region (**Figure 5A**). Alignment of the P88 tail fiber protein with that of pro147 showed that there were two segments of hypervariable regions of 120aa (576–695 in P88) and 31 aa (716–746 in P88) in the C-terminal region (**Figure 7A**).

Structure prediction and homology modeling of the tail fiber proteins of P88 and pro147 using the Phyre2 server^2^ showed that the C-terminal domain (amino acids 426–612) of the P88 tail fiber (**Figure 5B2**) had the same folding topology as that of the domain (amino acids 937–1127) of T4 phage gp34 (**Figure 5B1**), with confidence of 97.17%; the C-terminal domain (amino acids 423–546) of the pro147 tail fiber (**Figure 5B4**) had the same folding topology as that of the domain (amino acids 984–1108) of T4 phage gp34 (**Figure 5B3**), with confidence of 97.17%. Despite the high degree of structural similarity, the tail fiber domains of P88 (or pro147) and T4 phage gp34 shared low amino acid identity over the relevant sequences with identity of 18 and 21%, respectively (**Figure 5B**).

### Gene Mutants Were Constructed to Analyze the Key Regions and Sites

**FIGURE 6:**
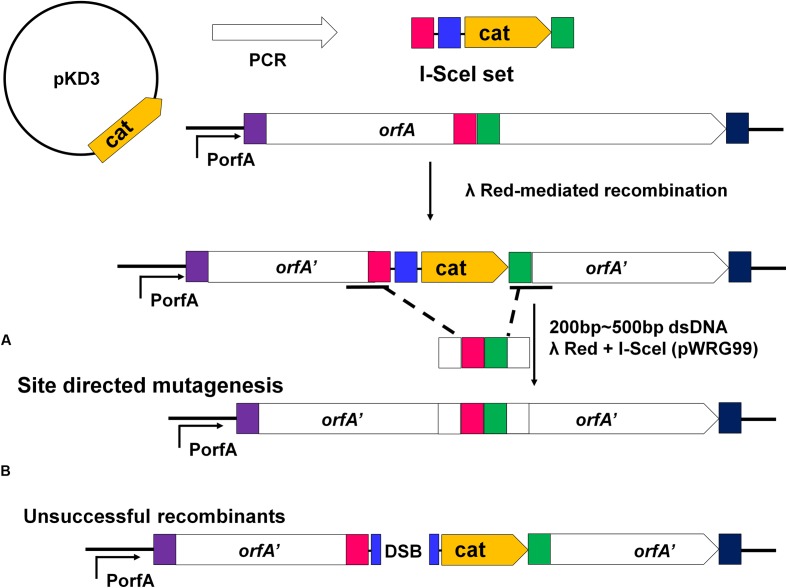
**Rationale of the recombineering method of scarless mutagenesis.** **(A)** chloramphenicol resistance cassette (cat) and an I-SceI recognition site were PCR amplified from plasmid pKD3 using primers with 40 bp homology extensions (red and green rectangles) and were integrated within a target gene “orfA” via Red-mediated recombination. The plasmid pWRG99 (containing the lambda Red recombinase and I-SceI endonuclease) and a 200–500 bp dsDNA homologous recombination fragment were transformed by electroporation sequentially. **(A)** Site-directed mutagenesis of the region adjacent to the I-SceI recognition site and the resistance cassette. With the help of Red-mediated recombination, homologous recombination occurred between the 200 and 500bp dsDNA fragment harboring the same homologous regions (red and green rectangles) and the corresponding sequence of K88. **(B)** The bacteria would be killed if homologous recombination did not occur. The I-SceI endonuclease expressed from plasmid pWRG99 would kill unsuccessful recombinants by induction of double-strand breaks.

**FIGURE 7:**
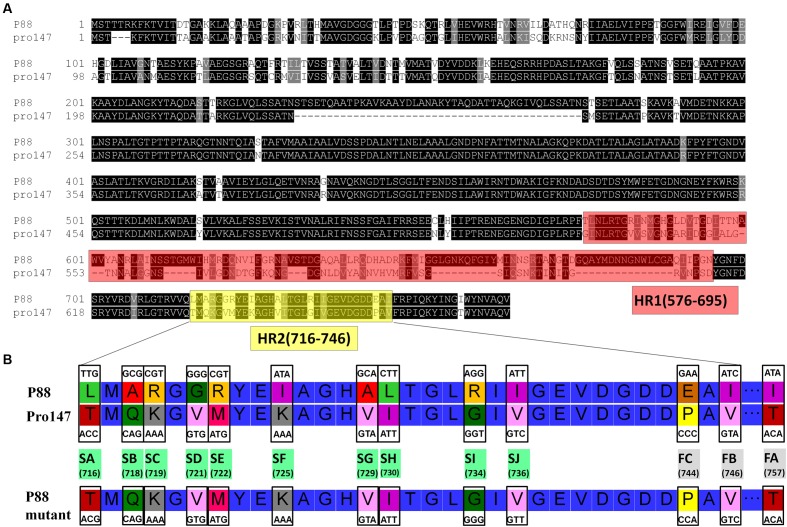
**Protein fragments and amino acids of tail fiber used for exchange mutagenesis.** **(A)** Protein fragments of the tail fiber for exchange mutagenesis. Alignment of P88 tail fiber protein with that from phages pro147 showed that there were two hypervariable regions of 120aa (576–695 in P88) and 31 aa (716–746 in P88) in the C-terminal region. Hypervariable region of 120aa (HR1) and the region of 31 aa (HR2) of P88 were replaced with the corresponding parts of pro147. **(B)** Amino acids of the tail fiber for exchange mutagenesis. Twelve different amino acids in the HR2 region (amino acid sets 716, 718, 719, 721, 722, 725, 729, 730, 734, 736, 744, and 746) and amino acid 757 of P88 were replaced with the corresponding amino acids of pro147.

Construction of gene mutants was carried out by modifying the phage genome in the genome of lysogenic bacteria and then inducing the production of the prophage (**Figure 2B**). The scarless mutagenesis method (**Figure 6**) was used to produce mutants of P88. We modified the tail fiber protein of P88 by replacing different parts of it with the corresponding parts of pro147. After tail fiber comparison of P88 and pro147, hypervariable regions of amino acids 576–695 (HR1) and 716–746 (HR2) (**Figure 7A**) in the C-terminus of the P88 tail fiber protein were replaced with the corresponding parts of pro147. After replacement of HR2, the phage mutant P88HR2 displayed a different host range to that of P88; therefore the 12 different amino acids in the region of HR2 [amino acid sets SA (716), SB (718), SC (719), SD (721), SE (722), SF (725), SG (729), SH (730), SI (734), SJ (736), FC (744), and FB (746)] (**Figure 7B**) and amino acid set FA (757) (**Figure 7B**) of P88 were replaced with the corresponding amino acids of pro147. Finally, nine recombinant bacteria of K88HR1 (576–695), K88HR2 (716–746), K88SD (721), K88SE (722), K88SH (730), K88SI (734), K88FA (757), K88FB (746) and K88FC (744) were constructed (**Table 2**).

**Table 2:** Results of the mutation experiment using scarless mutagenesis.

Name of DNA fragment or amino acid site	Corresponding amino acids sequence in P88	Mutant bacteria	Infect host DE048 of P88	Name of phage mutants
HR1	576–695	K88 HR1	Yes	P88 HR1
HR2	716–746	K88HR2	Yes	P88HR2
SD	721	K88SD	Yes	P88SD
SE	722	K88SE	Yes	P88SE
SH	730	K88SH	Yes	P88SH
SI	734	K88SI	Yes	P88SI
FA	757	K88FA	Yes	P88FA
FB	746	K88FB	Yes	P88FB
FC	744	K88FC	No	NA


### Induction and Lytic Capacity Analysis of P88 Mutants

**FIGURE 8:**
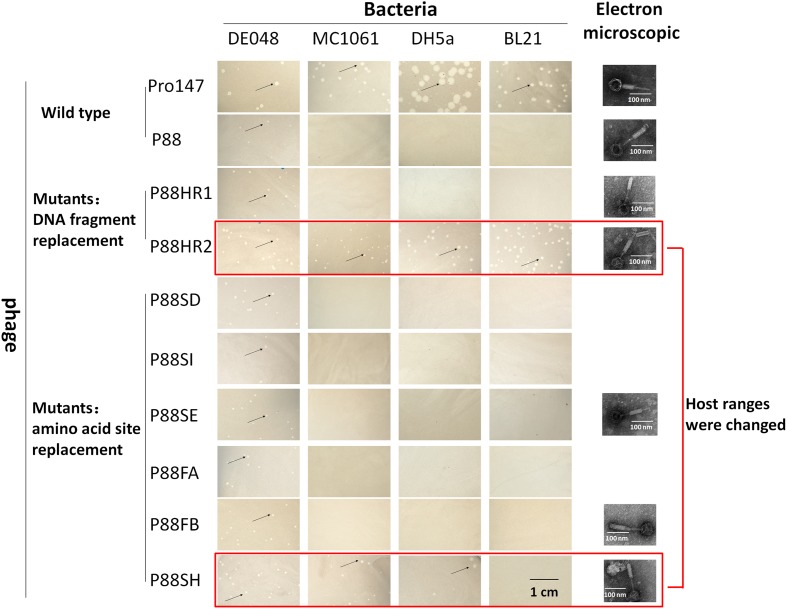
**Host range of P88, pro147 and P88 mutants (P88HR1, P88HR2, P88SD, P88SE, P88SH, P88SI, P88FA, and P88FB).** The host ranges of P88, pro147, and P88 mutants (P88HR1, P88HR2, P88SD, P88SE, P88SH, P88SI, P88FA, and P88FB) were displayed. On the lawn of DE048, all the phages could form clear plaques; on the lawn of MC1061 and DH5a, phages pro147, P88HR2, and P88SH could form clear plaques; on the lawn of BL21, phages pro147, and P88HR2 could form clear plaques. Phages pro147 and P88HR2 had the same host ranges.

The mutant bacteria were then induced by mitomycin C to evaluate their lysis capacity. Nine mutants of P88 were investigated. Eight mutants of K88HR1(576–695), K88HR2 (716–746), K88SD (721), K88SE (722), K88SH (730), K88SI (734), K88FA (757), and K88FB (746) could be induced to produce phages (P88HR1, P88HR2, P88SD, P88SE, P88SH, P88SI, P88FA, and P88FB) those were able to infect DE048 (**Table 2**; **Figure 8**), the host of P88; however, the bacteria mutant K88FC (744: glutamic acid to proline) could not be induced to produce phage that infected DE048 (**Table 2**). Meanwhile, K88FC could not be induced to produce phage to infect MC1061, DH5a, and BL21. The phage particles induced from the mutants that could lyse the *E. coli* strain DE048 were enriched for the subsequent experiment of host range determination and electron microscopy.

### The Host Range Test of P88 Mutants

The host ranges of P88, pro147 and P88 mutants (P88HR1, P88HR2, P88SD, P88SE, P88SH, P88SI, P88FA, and P88FB) were analyzed. After incubation at 37°C for 10 h, on the lawn of DE048, all the phages could form clear plaques; on the lawn of MC1061 and DH5a, phages pro147, P88HR2 and P88SH (730: leucine to isoleucine) could form clear plaques; on the lawn of BL21, phages pro147 and P88HR2 could form clear plaques; pro147 and P88HR2 had the same host ranges (**Figure 8**). P88, pro147 and all the P88 mutants could not infect DE048 when incubated at 28°C.

Electron micrographs showed that compared with those of the original P88, the head and the tail of the phage mutants with altered host ranges (P88HR2 and P88SH) and with no change in host range (P88HR1, P88SE, and P88FB) did not have visible differences (**Figure 8**).

## Discussion

We screened two P2-like coliphages, named P88 and pro147, and identified their corresponding host bacteria. Analysis of the key regions and sites that might determine receptor recognition showed that the host range of P88 was altered after replacing the hypervariable regions HR2 (amino acids 716–746) of the tail fiber protein of P88 with that of pro147; the host ranges of P88 with replacement of amino acid 730 (leucine) and 744 (glutamic acid) were changed after replacing six amino acids in HR2 with the corresponding amino acids of pro147. These results showed that, in P88, the tail fiber determined the receptor recognition; the region of amino acids 716–746 was important for receptor recognition; amino acids 730 (leucine) and 744 (glutamic acid) were the key amino acids of P88 that determine the host range.

Replacing regions of the tail fiber protein in P88 with those of pro147 changed the host range, which indicated that the corresponding regions of pro147 would determine its receptor recognition. Furthermore, the high homology of the tail fiber sequences in P2-like phages P88, pro147, P2 (NC_001895.1), and Wphi (005056.1) caused us to speculate that the corresponding parts in P2 and Wphi would also determine their receptor recognition specificity.

For temperate phages, when the phage genome is modified by manipulating the bacterial genome, the mutant strains constructed could be confirmed by PCR and the prophage could then be introduced and characterized. If the phage genome was modified in the host cell when it was separated from the bacterial chromosome by homologous recombination with a homologous fragment in the form of plasmid or DNA fragment, like lytic phages (Yoichi et al., 2005; Mahichi et al., 2009; Thomason et al., 2009), a plaque assay was used to screen the mutant phages; however, the recombinant phage would not be selected without a sensitive host strain. Hence, to construct mutants of temperate phages, modifying the phage genome by manipulating the bacterial genome was preferred.

The lysogeny module is required to form stable lysogens (Court et al., 2007). It is thought that a phage should be lytic and not lysogenic for use as an antibacterial agent (Chan et al., 2013). Here, the phage modification experiment was conducted on lysogenic bacteria (phage in the lysogenic state). In other words, the induced phage was temperate and able to perform lysogenic infections. Therefore, to disrupt lysogenic infection, the genes of lysogeny module could be deleted without disrupting the lytic ability of the phage (Zhang et al., 2013). Thus, compared with wild temperate phage strains, the novel modified phages are expected to minimize potential hazards to be integrated into the bacteria in normal flora if used as antibacterial agents.

In the next step of our research, we could create a homologous sequence library that contains sufficient allelic sequences to modify specific binding sequences by gene synthesis and homologous sequences enriched from natural samples. The crystal structure of the tail fiber protein with the key sites for receptor binding would be analyzed to understand how P2-like phages recognize their host.

## Conclusion

This study located the specific binding sequences that determined the phage host range of P88 and increased our understanding of the function of the tail fibers in P2-like phages. The result revealed that lysogenic bacteria could be used as vectors to change or broaden a phage’s host range. It might also be possible to modify the genomes to analyze gene function or change/expand the host range of other bacteriophages using this method.

## Author Contributions

WZ and MC conceived the study and designed experiments. MC, HY, and CL performed the data analysis. MC, LZ, and SX carried out experiments. MC and LZ analyzed experimental results. MC and WZ wrote the manuscript.

## Conflict of Interest Statement

The authors declare that the research was conducted in the absence of any commercial or financial relationships that could be construed as a potential conflict of interest.

## Footnotes

1 http://www.sbg.bio.ic.ac.uk/phyre2/html/
