# Supplementary material for: Inducible Prophage Mutant of Escherichia coli Can Lyse New Host and the Key Sites of Receptor Recognition Identification
Source: Front Microbiol. 2017 Feb 1;8:147. doi: 10.3389/fmicb.2017.00147 (PMC5285337; doi:10.3389/fmicb.2017.00147)
Supplement: Supplementary file 1 [file Table_1.DOC]

**Table S1** *E. coli* strains used in prophage induction experiments

| Strain | Place of isolation | Strain | Place of isolation |
| --- | --- | --- | --- |
| DE001 | Lishui, Jiangsu | DE132 | Bengbu, Anhui |
| DE008 | Lishui, Jiangsu | DE147 | Shandong |
| DE013 | Lishui, Jiangsu | DE148 | Shandong |
| DE018 | Lishui, Jiangsu | DE152 | Wuhan, Hubei |
| DE031 | Hexian, Anhui | DE160 | Lianyungang, Jiangsu |
| DE032 | Hexian, Anhui | DE169 | Jiangning, Jiangsu |
| DE044 | Hexian, Anhui | DE182 | Quanjiao, Anhui |
| DE054 | Lianyungang, Jiangsu | DE183 | Quanjiao, Anhui |
| DE056 | Lianyungang, Jiangsu | DE282 | Jiangning, Jiangsu |
| DE060 | Lianyungang, Jiangsu | DE283 | Jiangning, Jiangsu |
| DE061 | Lianyungang, Jiangsu | DE296 | Jiangning, Jiangsu |
| DE064 | Lianyungang, Jiangsu | DE303 | Jiangning, Jiangsu |
| DE072 | Qiligang, Jiangsu | DE312 | Jiangning, Jiangsu |
| DE075 | Qiligang, Jiangsu | DE316 | Jiangning, Jiangsu |
| DE077 | Qiligang, Jiangsu | DE327 | Jiangning, Jiangsu |
| DE096 | Liuhe, Jiangsu | DE384 | Jiangning, Jiangsu |
| DE098 | Liuhe, Jiangsu | DE389 | Jiangning, Jiangsu |
| DE101 | Liuhe, Jiangsu | DE402 | Jiangning, Jiangsu |
| DE102 | Liuhe, Jiangsu | DE414 | Jiangning, Jiangsu |
| DE104 | Liuhe, Jiangsu | DE419 | Jiangning, Jiangsu |
| DE119 | Liuhe, Jiangsu | DE432 | Chuzhou, Anhui |
| DE120 | Liuhe, Jiangsu | DE456 | Chuzhou, Anhui |
| DE123 | Liuhe, Jiangsu | DE458 | Chuzhou, Anhui |
| K88 | Guangzhou, Guangdong | RS218 | USA |
| O138 | Yangzhou, Jiangsu | NT01 | Nantong, Jiangsu |
| E1102 | Yangzhou, Jiangsu | O157 | (ATCC43889) |
| HX01 | Hexian, Anhui | MG1655 | (ATCC 47076) |
