# Supplementary material for: Inducible Prophage Mutant of Escherichia coli Can Lyse New Host and the Key Sites of Receptor Recognition Identification
Source: Front Microbiol. 2017 Feb 1;8:147. doi: 10.3389/fmicb.2017.00147 (PMC5285337; doi:10.3389/fmicb.2017.00147)
Supplement: Supplementary file 2 [file Table_2.DOC]

**Table S2** *Escherichia coli* strains used for phage isolation

| Strain | Place of isolation | Strain | Place of isolation |
| --- | --- | --- | --- |
| DE001 | Lishui, Jiangsu | DE152 | Wuhan, Hubei |
| DE002 | Lishui, Jiangsu | DE160 | Guannan, Jiangsu |
| DE003 | Lishui, Jiangsu | DE167 | Jiangning, Jiangsu |
| DE005 | Lishui, Jiangsu | DE169 | Jiangning, Jiangsu |
| DE007 | Lishui, Jiangsu | DE172 | Jiangning, Jiangsu |
| DE008 | Lishui, Jiangsu | DE182 | Quanjiao, Anhui |
| DE010 | Lishui, Jiangsu | DE183 | Quanjiao, Anhui |
| DE011 | Lishui, Jiangsu | DE186 | Jiangning, Jiangsu |
| DE013 | Lishui, Jiangsu | DE192 | Quanjiao, Anhui |
| DE015 | Lishui, Jiangsu | DE197 | Wuhu, Anhui |
| DE017 | Lishui, Jiangsu | DE205 | Bengbu, Anhui |
| DE018 | Lishui, Jiangsu | DE207 | Jiangning, Jiangsu |
| DE019 | Hexian, Anhui | DE209 | Jiangning, Jiangsu |
| DE021 | Hexian, Anhui | DE217 | Jiangning, Jiangsu |
| DE022 | Hexian, Anhui | DE241 | Quanjiao, Anhui |
| DE023 | Hexian, Anhui | DE242 | Quanjiao, Anhui |
| DE031 | Hexian, Anhui | DE248 | Laian, Anhui |
| DE032 | Hexian, Anhui | DE257 | Laian, Anhui |
| DE034 | Hexian, Anhui | DE278 | Jiangning, Jiangsu |
| DE041 | Hexian, Anhui | DE282 | Jiangning, Jiangsu |
| DE044 | Hexian, Anhui | DE283 | Jiangning, Jiangsu |
| DE048 | Quanjiao, Anhui | DE295 | Jiangning, Jiangsu |
| DE049 | Quanjiao, Anhui | DE296 | Jiangning, Jiangsu |
| DE054 | Lianyungang, Jiangsu | DE301 | Jiangning, Jiangsu |
| DE056 | Lianyungang, Jiangsu | DE302 | Jiangning, Jiangsu |
| DE060 | Lianyungang, Jiangsu | DE303 | Jiangning, Jiangsu |
| DE061 | Lianyungang, Jiangsu | DE312 | Jiangning, Jiangsu |
| DE064 | Lianyungang, Jiangsu | DE316 | Jiangning, Jiangsu |
| DE069 | Liuhe, Jiangsu | DE322 | Jiangning, Jiangsu |
| DE071 | Qiligang, Jiangsu | DE327 | Jiangning, Jiangsu |
| DE072 | Qiligang, Jiangsu | DE353 | Jiangning, Jiangsu |
| DE075 | Qiligang, Jiangsu | DE365 | Jiangning, Jiangsu |
| DE077 | Qiligang, Jiangsu | DE373 | Jiangning, Jiangsu |
| DE083 | Qiligang, Jiangsu | DE376 | Jiangning, Jiangsu |
| DE096 | Liuhe, Jiangsu | DE379 | Jiangning, Jiangsu |
| DE098 | Liuhe, Jiangsu | DE384 | Jiangning, Jiangsu |
| DE101 | Liuhe, Jiangsu | DE389 | Jiangning, Jiangsu |
| DE102 | Liuhe, Jiangsu | DE402 | Jiangning, Jiangsu |
| DE104 | Liuhe, Jiangsu | DE404 | Jiangning, Jiangsu |
| DE119 | Liuhe, Jiangsu | DE407 | Jiangning, Jiangsu |
| DE120 | Liuhe, Jiangsu | DE414 | Jiangning, Jiangsu |
| DE123 | Liuhe, Jiangsu | DE419 | Jiangning, Jiangsu |
| DE126 | Bengbu, Anhui | DE426 | Chuzhou, Anhui |
| DE127 | Bengbu, Anhui | DE432 | Chuzhou, Anhui |
| DE132 | Bengbu, Anhui | DE452 | Chuzhou, Anhui |
| DE134 | Shandong | DE456 | Chuzhou, Anhui |
| DE137 | Shandong | DE458 | Chuzhou, Anhui |
| DE144 | Shandong | DE464 | Chuzhou, Anhui |
| DE147 | Shandong | RS218 | USA |
| DE148 | Shandong | NT01 | Nantong, Jiangsu |
| K88 | Guangzhou, Guangdong | MG1655 | (ATCC 47076) |
| O138 | Yangzhou, Jiangsu | O157 | (ATCC43889) |
| E1102 | Yangzhou, Jiangsu | MC1061 | (ATCC 53338) |
| DH5a | engineering bacteria | BL21 | engineering bacteria |
