# Supplementary material for: Inducible Prophage Mutant of Escherichia coli Can Lyse New Host and the Key Sites of Receptor Recognition Identification
Source: Front Microbiol. 2017 Feb 1;8:147. doi: 10.3389/fmicb.2017.00147 (PMC5285337; doi:10.3389/fmicb.2017.00147)
Supplement: Supplementary file 3 [file Table_3.DOC]

**Table S3** Primers and plasmids used in this study by recombineering method of scarless mutagenesis

Primers used in this study

| Primers purpose | Primer | Sequence (5’to 3’) | Corresponding amino acids sequence in P88 |
| --- | --- | --- | --- |
| Deletion primers | | | |
| Amplify I-SceI and resistance gene | k1-F | GTGTAGGCTGGAGCTGCTTC |  |
|  | k1-R | TTCTCTAGACTATATTACCCTGTTATCCCTAGCGTAACTAAGTATAGGAACTTCGGCGC |  |
|  |  |  |  |
| redHR1 | hk2-F | CTGAAGAATGCCTGCATATCATCCCTACACGAGAGAATGAGCTAGACTATATTACCCTGTTATCCCTA | 576-695 |
|  | hk2-Ｒ | CAACACGTGTGCCAAGTCGGACATCTCTCACATAGCGTGAGTCGCCTTACGCCCCGCCCTGC |  |
|  |  |  |  |
| redHR2 | cok2F | CTATGTGAGAGATGTCCGACTTGGCACACGTGTTGTTCAACTAGACTATATTACCCTGTTATCCCTA | 716-746 |
|  | cok2R | TATACCATATGCCATTGATGTATTTTTGTATCGGCCTGAACGCCTTACGCCCCGCCCTGC |  |
|  |  |  |  |
| Primers for amplifying fragments for recombination | | |  |
| HR1 | hk3F | CTGAAGAATGCCTGCATATCATCCCTACAC | 576-695 |
|  | hk3R | CAACACGTGTGCCAAGTCGGACATCTCTCAC |  |
|  |  |  |  |
|  | hk3F1extend | CGACAGTCAATGCACTGAGGATATTTAATTCATCTTTTGGTGCCATTTTTCGCCGTTCTGAAGAATGCCTGCATATC |  |
|  | hk3R1extend | CCGGTAAGTGCGTGTCCGGCTATTTCATAACGCCCTCCACGCGCCATCAATTGAACAACACGTGTGCCAAGTCGG |  |
|  |  |  |  |
|  | hk3F2extend | TGGGATGCTTTGTCTGTTCTTGTTAAAGCCCTTTTCAGCAGTGAAGTAAAAATATCGACAGTCAATGCACTGAGG |  |
|  | hk3R2extend | GTATCGGCCTGAAGATAGCTTCATCATCGCCATCAACTTCACCAATAATCCTTAATCCGGTAAGTGCGTGTCCGGC |  |
|  |  |  |  |
| HR2 | cok3F | CTATGTGAGAGATGTCCGACTTGGCACACGTG | 716-746 |
|  | cok3R | CACCTGTGCGACGTTATACCATATGCCATTG |  |
|  |  |  |  |
|  | Cok31F | GGACGTAATGCGGTATCCACTG |  |
|  | Cok31R(scr) | CACGTGTGCCAAGTCGGACATCTCTCACATAG |  |
|  |  |  |  |
|  | Cok33F(scr) | CAATGGCATATGGTATAACGTCGCACAGGTG |  |
|  | Cok33R | CCTTCAGGGTTAAGCGTGGAGGC |  |
|  |  |  |  |
| SA | SAF | GGGATGCTTTGTCTGTTCTTGTTAAAGCCC | 716 |
|  | SAR | CCAATAATCCTTAATCCGGTAAGTGCGTGTCCGGCTATTTCATAACGCCCTCCACGCGCCATCGTTTG |  |
|  |  |  |  |
| SB | SBF | GGGATGCTTTGTCTGTTCTTGTTAAAGCCC | 718 |
|  | SBR | CCAATAATCCTTAATCCGGTAAGTGCGTGTCCGGCTATTTCATAACGCCCTCCACGCTGCATC |  |
|  |  |  |  |
| SC | SCF | GGGATGCTTTGTCTGTTCTTGTTAAAGCCC | 719 |
|  | SCR | CAATAATCCTTAATCCGGTAAGTGCGTGTCCGGCTATTTCATAACGCCCTCCTTTCGC |  |
|  |  |  |  |
| SD | SDF | GGGATGCTTTGTCTGTTCTTGTTAAAGCCC | 721 |
|  | SDR | CACCAATAATCCTTAATCCGGTAAGTGCGTGTCCGGCTATTTCATAACGCACTCC |  |
|  |  |  |  |
| SE | SEF | GGGATGCTTTGTCTGTTCTTGTTAAAGCCC | 722 |
|  | SER | CTTCACCAATAATCCTTAATCCGGTAAGTGCGTGTCCGGCTATTTCATACATCCC |  |
|  |  |  |  |
| SF | SFF | GGGATGCTTTGTCTGTTCTTGTTAAAGCCC | 725 |
|  | SFR | CCATCAACTTCACCAATAATCCTTAATCCGGTAAGTGCGTGTCCGGCTTTTTC |  |
|  |  |  |  |
| SG | SGF | GGGATGCTTTGTCTGTTCTTGTTAAAGCCC | 729 |
|  | SGR | CATCGCCATCAACTTCACCAATAATCCTTAATCCGGTAAGTACGTG |  |
|  |  |  |  |
| SH | SHF | GGGATGCTTTGTCTGTTCTTGTTAAAGCCC | 730 |
|  | SHR | CTTCATCATCGCCATCAACTTCACCAATAATCCTTAATCCGGTAATTGC |  |
|  |  |  |  |
| SI | SIF | GGGATGCTTTGTCTGTTCTTGTTAAAGCCC | 734 |
|  | SIR | CCTGAAGATAGCTTCATCATCGCCATCAACTTCACCAATAATCCCTAATC |  |
|  |  |  |  |
| SJ | SJF | GGGATGCTTTGTCTGTTCTTGTTAAAGCCC | 736 |
|  | SJR | CGGCCTGAAGATAGCTTCATCATCGCCATCAACTTCACCAACAATC |  |
|  |  |  |  |
| FA | FAF | CAGCAGCACAGGCATGTGGATTCATATG | 757 |
|  | FAR | TTAAATGCTGCATAATTTACACCTGTGCGACGTTATACCATGTGCCATTGATG |  |
|  |  |  |  |
| FB | FBF | ATGGTCTGGATGTTACAGGAGATATAA | 746 |
|  | FBR | CCATATGCCATTGATGTATTTTTGTATCGGCCTGAAGACAGCTTCATCAT |  |
|  |  |  |  |
| FC | FCF | TCGACAGTCAATGCACTGAGGATA | 744 |
|  | FCR | GATGTATTTTTGTATCGGCCTGAAGATAGCTGGATCATCGCCATCAACTT |  |
|  |  |  |  |
| Control primers | | |  |
| Check recombination | Checkf | TGCCTGCATATCATCCCTACACGAG |  |
|  | Checkr | TCGTCTGCCGTATAAATCCGTTTAACCAC |  |
|  |  |  |  |
| Check redHR1 | H1F | CGCCCTTGTAGATTCGTCACC |  |
|  | H1R | CTTCCTTAGCTCCTGAAAATCTCGAC |  |
|  |  |  |  |
| Check redHR2 | C1F | GGAAACGACCCGAATTTTGCGACC |  |
|  | C1R | AACGGTGTAACAAGGGTGAACACTATCCCA |  |

Plasmids used in this study

| Plasmid | Characteristics |
| --- | --- |
| pKD46 | Amp; expresses lambda Red recombinase |
| pKD3 | cat gene; template plasmid |
| pWRG99 | Amp; expresses lambda Red recombinase; express I-SceI |
